# Effects of stress associated with academic examination on the kynurenine pathway profile in healthy students

**DOI:** 10.1371/journal.pone.0252668

**Published:** 2021-06-03

**Authors:** Kyaimon Myint, Kelly Jacobs, Aye Mu Myint, Sau Kuen Lam, Lyndal Henden, See Ziau Hoe, Gilles J. Guillemin

**Affiliations:** 1 Department of Physiology, Faculty of Medicine, University of Malaya, Kuala Lumpur, Malaysia; 2 Neuroinflammation Group, Department of Biomedical Sciences, Faculty of Medicine and Health Sciences, Macquarie University, Sydney, NSW, Australia; 3 Psychoneuroimmunology Research Group, European Collaborative Project, Munich, Germany; 4 Department of Pre-clinical Sciences, Faculty of Medicine and Health Sciences, Universiti Tunku Abdul Rahman, Bandar Sungai Long, Kajang, Malaysia; 5 Genomics and Bioinformatics Group, Department of Biomedical Sciences, Faculty of Medicine and Health Sciences, Macquarie University, Sydney, NSW, Australia; Lancaster University, UNITED KINGDOM

## Abstract

The effects of stress on the neuroendocrine, central nervous and immune systems are extremely complex. The kynurenine pathway (KP) of the tryptophan metabolism is recognised as a cross-link between the neuroendocrine- and immune systems. However, the effects of acute stress from everyday life on KP activation have not yet been studied. This study aims to investigate changes in the levels of the KP neuroactive metabolites and cytokines in response to stress triggered by academic examinations. Ninety-two healthy first year medical students benevolently participated in the study. Parameters were measured pre- examination, which is considered to be a high-stress period, and post-examination, as a low-stress period. Stress induced by academic examinations significantly increases the perceived stress scores (*p*<0.001), serum cortisol levels (*p*<0.001) and brain-derived neurotrophic factor (BDNF) levels (*p*<0.01). It decreased IL-10 levels (*p*<0.05) but had no effect on IL-6 and TNF-alpha levels. Only the KP neuroactive metabolite, 3-hydroxykynurenine (3-HK) significantly increased (*p*<0.01) in the post-examination period. In addition, the stress scores positively correlated with the levels of cortisol (*r*^2^ = 0.297, *p*<0.01) at post examination. Acute stress triggered by academic examinations increases cortisol and BDNF production and suppresses the anti-inflammatory cytokine, IL-10, but did not increase significantly the levels of other pro-inflammatory cytokines, tryptophan, kynurenine and downstream KP metabolites. The concomitant increased levels of BDNF under the duress of acute examination stress appear to limit the levels pro-inflammatory markers, which may attenuate the action of cortisol and the neuroinflammatory branch of the KP.

## Introduction

As stress is a prevalent element of the modern human life, it is therefore important to identify the underlying complex mechanisms involved in stress reactivity. The duration of stress is crucial in determining the nature of the stress-induced physiological changes; short-term stress triggers different kinds of neuro-immune responses, which can be either beneficial or detrimental whereas chronic or extreme stress always has a negative impact on physiological homeostasis and psychological adaptations of individuals.

Acute stress is well known to increase the activity of hypothalamo-pituitary adrenal (HPA) axis [1, 2]. Activation of the peripheral components of the HPA axis triggers the systemic production of glucocorticoids as an adaptive response to stress aiming to re-establish and maintain homeostasis [1, 2]. While the chronic stress increases the activity of the HPA axis, with subsequent alteration of the neuroendocrine system [1–3] as well as the immune functions and cytokine profile [4, 5]. The brain-derived neurotrophic factor (BDNF), whose primary role is in the process of neuroplasticity, is also involved in the stress circuitry. The evidence of chronic stress-related alteration in brain BDNF expressions indicates the co-existence of BDNF and glucocorticoid signalling pathways in the central nervous system, particularly in the hippocampus [6–10].

**Fig 1:**
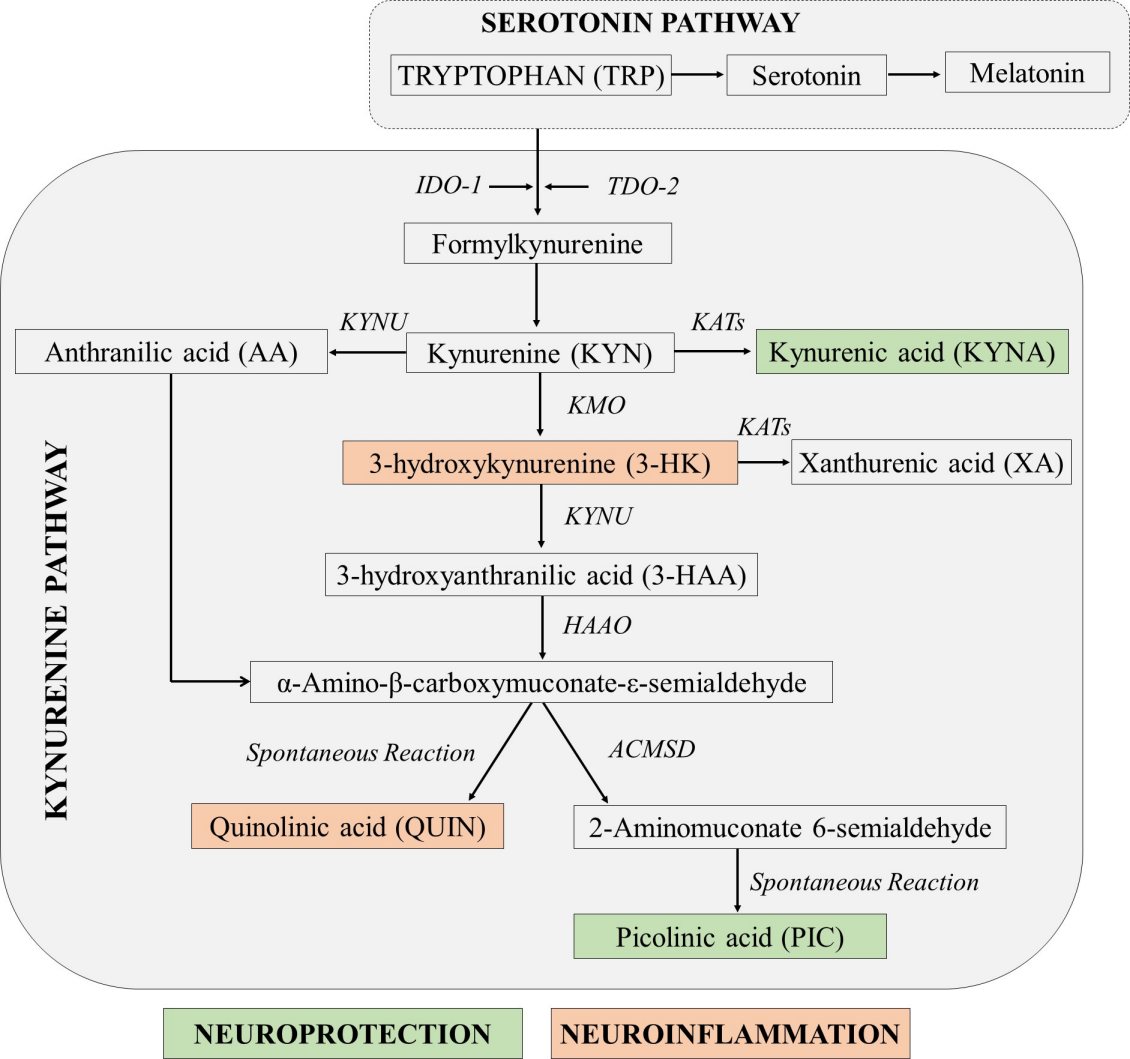
Simplified diagram of the kynurenine pathway [Modified from [15]].

A likely candidate able to orchestrate the complex interactions between central nervous-, neuroendocrine- and immune systems is the kynurenine pathway (KP) [11–13]. The KP is the main catabolic pathway for the essential amino acid tryptophan (TRP) [11–13] **(Fig 1)**. The various KP metabolites can have neuroprotective, immunomodulatory and /or neurotoxic functions [14–17]. Stress induces dysfunction of the neuroendocrine-immune system that subsequently activates the KP towards its neurotoxic branch ultimately leading to 3-hydroxykynurenine (3-HK) and quinolinic acid (QUIN) production [11, 12, 15–19]. The initial step for the activation of KP is the conversion of TRP to kynurenine (KYN) by two enzymes, tryptophan 2, 3-dioxygenase-2 (TDO-2) or indoleamine 2, 3-dioxygenase-1 (IDO-1). TDO-2 is induced by cortisol and is mostly found in liver, kidney and brain [20, 21]. IDO-1 is activated by pro-inflammatory mediators especially interferon (IFN)-γ and lipopolysaccharides [13, 14, 19, 22] and is present in numerous brain and peripheral cells including astrocytes, microglia, neurons, microvascular endothelial cells and macrophages [23–25]. Formation of first stable intermediate KYN is the key branching point between neuroprotective and neurotoxic branches of the KP **(Fig 1)**. Kynurenine is metabolised to produce neuroprotective kynurenic acid (KYNA) by the kynurenine aminotransferases (KATs); or into 3-HK, a neurotoxic and redox-active metabolite by kynurenine monooxygenase (KMO). The 3-HK is then metabolised to 1) anthranilic acid (AA) by kynureninase (KYNU), or 2) 3-hydroxyanthranilic acid (3-HAA) by 3- hydroxyanthranilic oxygenase (3-HAO) and ultimately to the neurotoxin quinolinic acid (QUIN) [16, 26]. These neurotoxins, 3-HK and QUIN are involved in the pathogenesis of most of the major neurodegenerative diseases [15, 27–29].

Among the different subtypes of stress, academic stress is considered to be the most common psychological stress among primary, secondary, undergraduate and postgraduate students. The degree of stress perceived may differ from the nature of the course taken but generally it is shown that medical students have higher stress levels during their training [30–33]. The total prevalence of stress varied in medical students, from 29.6% [34] to more than 90% [35], and academic examinations are revealed to be the most powerful acute stressors.

There have been substantial research done on the consequences of academic examination-induced stress on neuroendocrine and immune parameters. Although acute stress has been shown to increase the activity of HPA axis [1–3], the results of acute examination stress on cortisol secretion is inconsistent [36–41]. In addition, an altered Th1/Th2 balance was found between pre- and post-examination periods in exam stress model of medical students [42, 43].

Most of studies have looked at the consequences of chronic stress-induced changes on BDNF and KP neuroactive metabolites. However, the potential effects of acute stress on BDNF and KP metabolites levels remain uncovered. Thus, the present study aimed to determine the effects of academic stress triggered by examination on circulating neuro-endocrine-immunological mediators such as kynurenine metabolites, hormones and cytokines in healthy undergraduate medical students.

## Materials and methods

The ethical approval (MEC Ref. No. 781.12) was obtained by the Human Ethics Committee of the University Malaya Medical Centre (UMMC) Malaysia prior to the commencement of the study. The study was not pre-registered.

### Recruitment of participants

The participants were recruited from first-year medical students at The University of Malaya, Kuala Lumpur, Malaysia. A call for volunteer-based participation was made to around 140 students during their routine small group teaching classes and those who responded were requested to complete a questionnaire on their past and present medical history, personal information, socioeconomic status and lifestyle. Sample size was not predetermined. The following inclusion criteria were applied: no histories of acute illnesses, no previous known medical conditions or psychosocial problems, no financial constraints. All selected students met the inclusion criteria. Detailed information about the purpose, procedures and prerequisites of the study were provided to all participants prior to the day of experiment and their written consent was obtained. An honorarium was given for their participation in the study.

### Experimental design

The study was performed at two time-points, 1) pre-examination period: 1 or 2 days before; and 2) post-examination period: 1 week after the final written academic examination when the students were on holidays while waiting for the results. The examination lasted for 4 days and consisted of written theory as well as objective structured practical examination on basic medical science subjects. The first time-point (pre-examination period) was considered to be a high-stress setting and the second time-point (post-examination period) was considered to be a low-stress setting. The determination of perceived stress levels and collection of blood for analysis of biochemical parameters were carried out at both time-points. The students were requested to avoid exercises on the day of data collection. Each participant remained anonymous as all blood samples and matching questionnaires were coded with numbers.

### Subjective assessment: Stress questionnaire

A 42-item self-report validated depression, anxiety, stress scales (DASS) was provided to assess the levels of stress as perceived by the participants. This stress questionnaire is designed to measure the three related negative emotional states of depression, anxiety and tension/stress [44]. After brief introduction of DASS, the participants were asked to complete the 4-point severity/frequency scales to rate the extent to which they have experienced each state over the past week. In this study, only the stress scales were used.

### Objective assessment: Biochemical analysis

Blood samples were collected from all participants between 8:00 am to 9:00 am after a brief relaxation period of approximately 15 minutes. The samples were collected in vacutainers; sera were separated after centrifuging at 1000 x g and 4°C for 20 minutes and then kept at −80°C until the time of analysis. All the samples were assayed at the same time for determination of each parameter to limit experimental variability.

The levels of neuroendocrine hormone, cortisol in the serum were measured by chemiluminescent immunoassays (ADVIA Centaur, USA) at the Clinical Diagnostic Laboratory, University Malaya Medical Centre.

The serum concentrations of the neurotrophin BDNF and cytokines, IL-6, IL-10, and TNF-α were determined using ProcartaPlex Multiplex Immunoassay at i-DNA laboratory in Kuala Lumpur, Malaysia. A four-parameter logistics model was used to calculate the sample concentrations by interpolation. Cytokines under the limit of detection were set to the values equivalent to lower limit of quantitation for statistical analysis.

The quantification of the KP metabolites was performed at the Motor Neuron Disease (MND) Research Centre, Faculty of Medicine and Health Sciences, Macquarie University, Australia. The concurrent quantification of serum TRP, KYN, AA, 3-HK and 3-HAA serum levels was carried out using ultra high performance liquid chromatography (uHPLC), according to our previously published method [45] with slight modification [46].

### Statistical analysis

The statistical analysis was performed using GraphPad Prism software version 5.1 and version 9.1.0.

The perceived stress scores were calculated by adding the scores for the 14-relevant items in DASS scales for stress.

All blood samples were analysed in duplicate, and the mean value was calculated for each parameter and used for statistical analysis. After testing the normality of data by the Shapiro–Wilk test, a non-parametric Wilcoxon signed-rank test was used for comparison of quantitative variables of two time-points serum concentrations. Results are expressed as median and interquartile range (IQR). The gender difference between the parameters was determined by Shapiro–Wilk test for normality of the data. For those data that are not normally distributed, Mann Whitney test was used, and the results are expressed in median (IQR). The *t* test with Welch’s correction was used for calculation of means±SD in normally distributed data.

The potential correlations between the parameters were determined using Spearman correlation matrix.

## Results

A total of 92 (46 males, 46 females; 54% Malay, 43% Chinese and 3% Indians) healthy first-year medical students with the mean age of 19.96±0.42 and body mass index (BMI) of 21.63±3.21 were recruited for this study. All participants were single, neither smokers nor drinkers, and were free from medication and financial constraints. Since they were institutionalised in the hostels, most of their diets were assumed to be standardised.

The DASS-stress scores are higher (*p* < 0.001) in pre-examination period than that of post-examination period **(Table 1A)**. The levels of serum cortisol (*p* < 0.001) and BDNF (*p* < 0.01) are significantly increased in the pre-examination period compared to the post-examination period **(Table 1B)**.

**Table 1:** Changes in a) perceived stress scores, b) the levels of cortisol and BDNF, c) concentrations of cytokines, and d) concentrations of TRP and KP metabolites during pre-examination and post-examination period.

	Pre-examination period	Post-examination period	Fold changes	*p* value
	Median (IQR); n = 92	Median (IQR); n = 92	Pre/Post	
**a) Perceived stress scores**				
DASS-stress scores (normal score: 0−10)	11.00 (15.00–7.25)	7.00 (12.00–3.00)	↓ 1.57	*<*0.001***
**b) Levels of cortisol and BDNF**				
Cortisol (nmol/L)	465.5 (528.5–389.3)	421.0 (494.5–365.5)	↓ 1.1	*<*0.001***
BDNF (pg/mL)	520.4 (1361.0–73.80)	254.7 (921.4–41.52)	↓ 2.04	*<*0.01**
**c) Concentrations of cytokines**				
IL-10 (pg/mL)	0.63 (1.11–0.24)	0.81 (1.29–0.36)	↑ 1.29	*<*0.05*
IL-6 (pg/mL)	0.24 (0.72–0.24)	0.20 (1.24–0.01)	↔ 1.2	0.757; ns
TNF-α (pg/mL)	1.0 (1.52–0.58)	1.06 (1.65–0.58)	↔1.06	0.886; ns
**d) Concentrations of TRP and KP metabolites**				
TRP (μM)	37.55 (42.54–30.23)	35.75 (41.60–29.52)	↔1.05	0.316; ns
KYN (μM)	1.29 (1.46–1.07)	1.31 (1.51–1.12)	↔1.02	0.707; ns
3-HK (nM)	52.27 (63.64–41.01)	59.09 (68.18–50.00)	↔1.13	*<*0.01**
3-HAA (nM)	22.73 (28.78–16.53)	21.84 (28.63–17.97)	↔1.04	0.835; ns
AA (nM)	45.69 (57.12–37.53)	44.14 (61.91–36.24)	↔1.04	0.651; ns
KYN:TRP	34.75 (39.57–29.45)	34.04 (41.66–31.56)	↔1.02	0.574; ns
3-HAA:AA	0.47 (0.72–0.30)	0.49 (0.72–0.35)	↔1.04	0.963; ns

↑: Increased; ↓: decreased, ↔: Unchanged; ns: No significant difference.

The cytokine profile is shown in **Table 1C**. The level of anti-inflammatory cytokine, IL-10 is significantly reduced (*p* < 0.05) during the pre-examination period compared to post-examination period, while no significant change is observed in pro-inflammatory cytokines, IL-6 and TNF-α.

The serum levels of the KP metabolites for both time-points are shown in **Table 1D**. Except for the significantly increased 3-HK levels (*p* < 0.01) during the post-examination period, the levels of TRP and KYN have not significantly changed between the time-points. In addition, no significant changes were observed with the other metabolites, 3-HAA, AA or the KYN/TRP and 3-HAA/AA ratio.

The effects of gender differences on measured parameters are shown in Table 2. As compared to males, females showed significantly higher levels of DASS-perceived stress scores (*p* < 0.01) during pre-examination period (**Table 2A**), and BDNF (*p* < 0.05) during both pre- and post-examination periods (**Table 2B**). Meanwhile, the levels of TNF-α were significantly higher in males at post-examination. In addition, significantly higher levels of 3-HK (*p* < 0.05) in females during pre-examination period (**Table 2D**), and 3-HAA/AA ratio in males (*p* < 0.05) (**Table 2D**) during post-examination period were observed.

**Table 2:** Gender differences in a) perceived stress scores, b) the levels of cortisol and BDNF, c) concentrations of cytokines, and d) concentrations of TRP and KP metabolites during pre-examination and post-examination period.

	Pre-examination period		Post-examination period	
	Median (IQR)^#^/Mean [±SD]^##^		Median (IQR)^#^/Mean [±SD]^##^	
	Males; n = 46	Females; n = 46	Males; n = 46	Females; n = 46
**a) Perceived stress scores**				
DASS-stress scores	9.0 (14.0–6.0)	13.0 (16.0–10.0)**	5.5(9.25–2.0)	9.0 (12.0–4.0)
**b) Levels of cortisol and BDNF**				
Cortisol (nmol/L)	461.5 (515.3–377.0)	490.0 (558.8–400.5)	437.20 [±82.81]	416.90 [±103.6]
BDNF (pg/mL)	185.7 (1077.0–52.79)	918.4 (1440.0–140.7)*	96.61 (892.9–23.22)	559.0 (1259.0–79.71)*
**c) Concentrations of cytokines**				
IL-10 (pg/mL)	0.75 (1.29–0.24)	0.52 (1.05–0.23)	0.81 (1.42–0.43)	0.78 (1.13–0.22)
IL-6 (pg/mL)	0.24 (0.72–0.24)	0.24 (0.72–0.20)	0.20 (1.03–0.01)	0.25 (1.24–0.02)
TNF-α (pg/mL)	0.75 (1.29–0.24)	0.52 (1.05–0.23)	1.36 (1.65–0.58)	0.78 (1.36–0.54) *
**d) Concentrations of TRP and KP metabolites**				
TRP (μM)	38.24 (42.43–31.13)	37.25 (42.68–28.09)	37.43 (43.28–30.68)	33.22 (38.72–29.14)
KYN (μM)	1.32 [±0.29]	1.24 [±0.32]	1.35 (1.56–1.16)	1.25 (1.43–1.01)
3-HK (nM)	47.73 (61.93–36.96)	56.52 (63.64–49.43) *	59.09 (68.18–47.73)	61.12 (68.75–52.27)
3-HAA (nM)	22.02 (26.77–16.64)	23.71 (31.88–15.50)	23.02 (29.59–19.45)	20.66 (28.04–15.65)
AA (nM)	43.09 (51.71–36.89)	48.13 (63.99–37.54)	43.49 (53.32–34.63)	45.10 (75.07–37.21)
KYN:TRP	34.99 (39.65–30.52)	34.18 (39.63–29.05)	34.14 (40.11–31.75)	33.54 (43.19–31.43)
3-HAA:AA	0.51 (0.73–0.32)	0.44 (0.72–0.28)	0.51 (0.74–0.42)	0.46 (0.68–0.19)*

* As compared to Female in respective time-periods. * *p* < 0.05,

** *p* <0.01

^#^Median (IQR) for not normally distributed data

^##^Mean [±SD] for normally distributed data

### Correlational analyses

**Fig 2:**
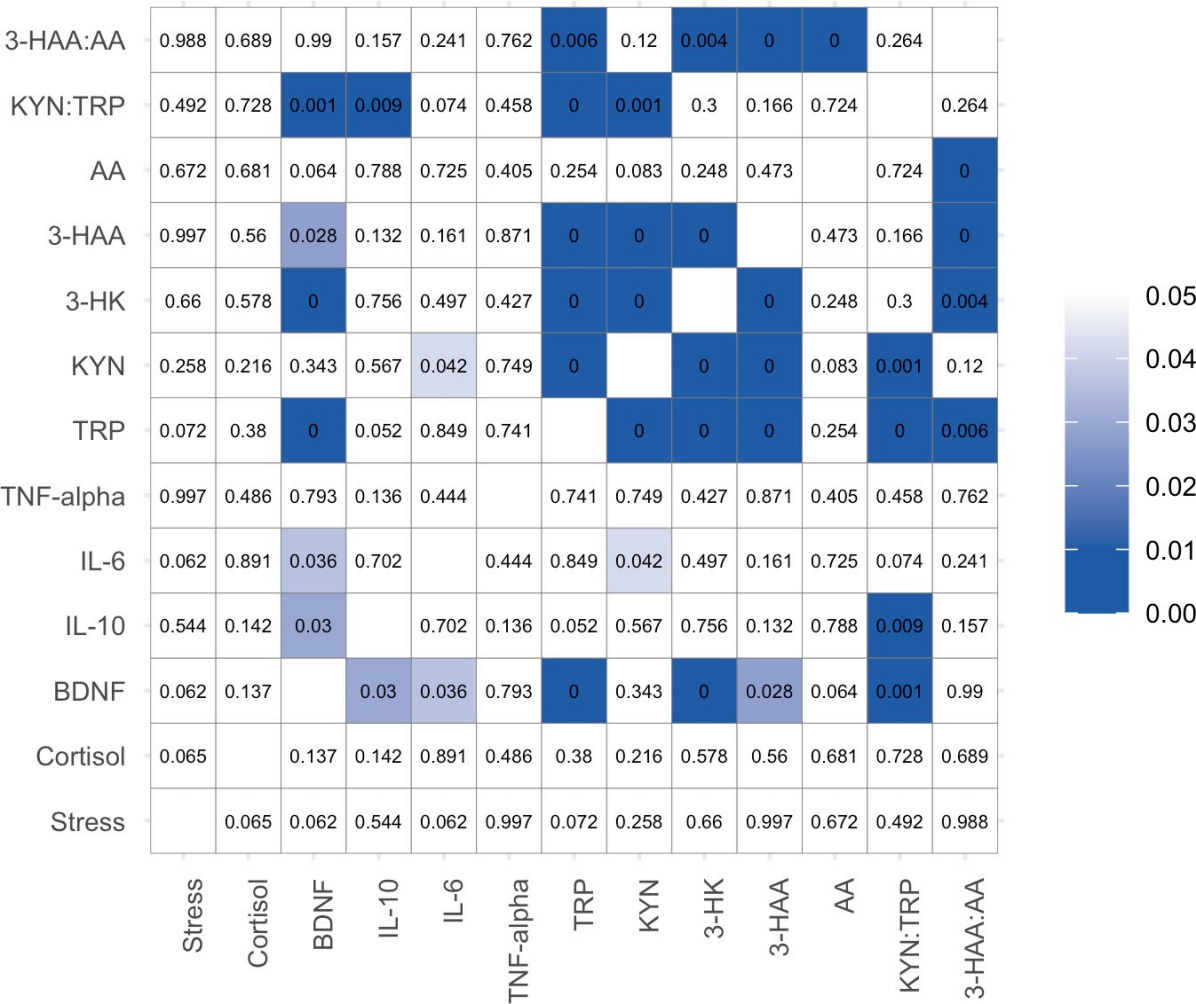
Correlation matrix showing *p* values between perceived stress scores, the levels of cortisol and BDNF, cytokines, and TRP and KP metabolites during pre-examination period.

During the pre-examination period, no significant correlations were found between the DASS-stress scores and other parameters, cortisol, BDNF, pro- and anti- inflammatory cytokines and KP metabolites. Serum levels of BDNF are found to be correlated with IL-10 (*r*^2^ = - 0.227, *p*<0.05), IL-6 (*r*^2^ = 0.219, *p*<0.05), TRP (*r*^2^ = 0.459, *p*<0.001), 3-HK (*r*^2^ = 0.501, *p*<0.001), 3-HAA (*r*^2^ = 0.229, *p*<0.05) and KYN:TRP (*r*^2^ = -0.336, *p*<0.001). The levels of KP metabolite TRP were significantly associated with BDNF (*r*^2^ = 0.459, *p*<0.001), KYN (*r*^2^ = 0.588, *p*<0.001), 3HK (*r*^2^ = 0.637, *p*<0.001), 3HAA (*r*^2^ = 0.509, *p*<0.001), KYN:TRP (*r*^2^ = - 0.499, *p*<0.001) and 3-HAA:AA (*r*^2^ = 0.285, *p*<0.01) (**Fig 2)**.

**Fig 3:**
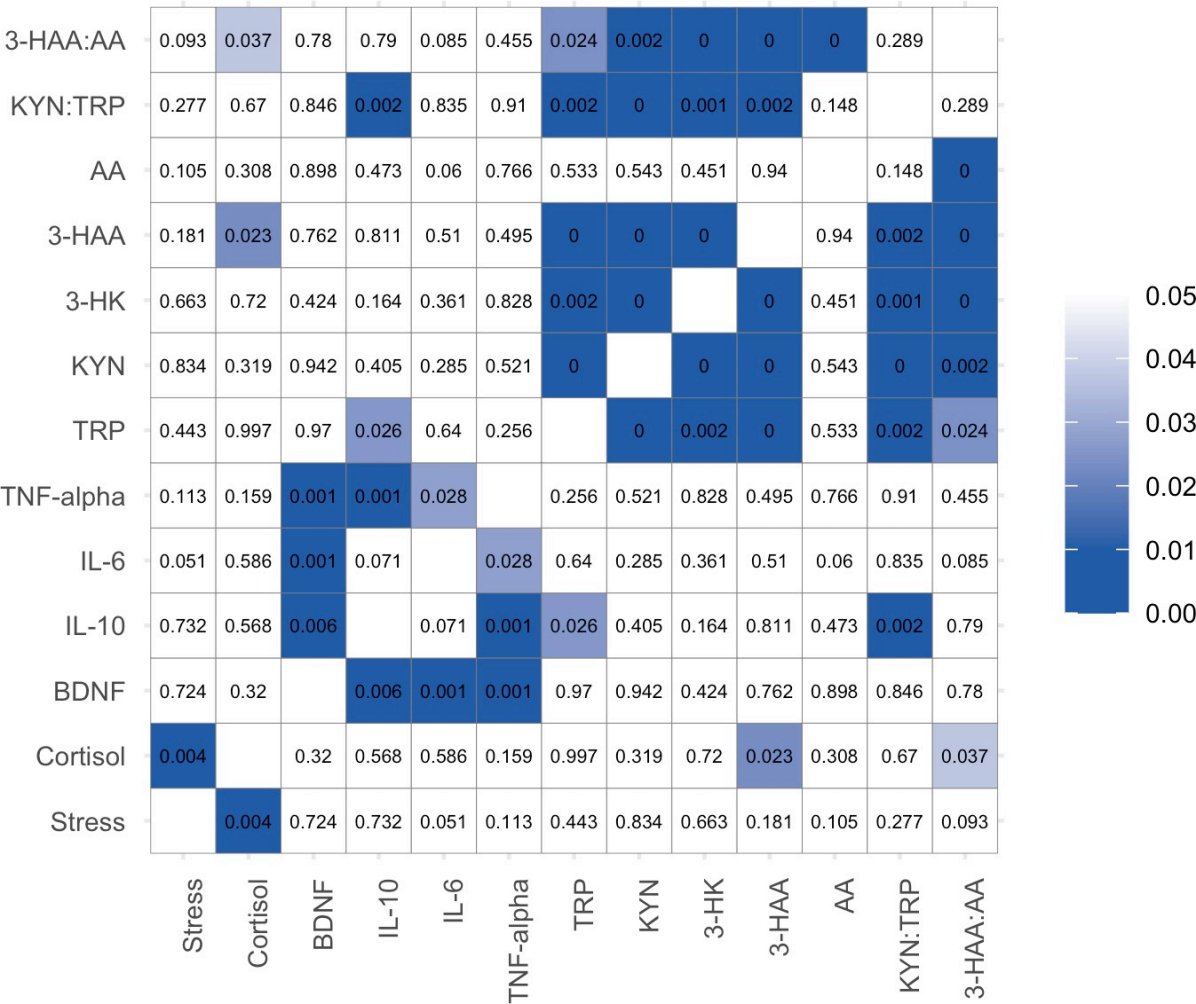
Correlation matrix showing *p* values between perceived stress scores, the levels of cortisol and BDNF, cytokines, and TRP and KP metabolites during post-examination period.

However, during the post-examination period, correlations were found between DASS-stress scores and the levels of cortisol (*r*^2^ = 0.297, *p*<0.01); levels of cortisol with 3-HAA (*r*^2^ = - 0.237, *p*<0.05) and 3-HAA:AA (*r*^2^ = - 0.176, *p*<0.05); BDNF with IL-10 (*r*^2^ = - 0.286, *p*<0.01), IL-6 (*r*^2^ = 0.331, *p*<0.001) and TNF-α (*r*^2^ = - 0.331, *p*<0.001); TRP with IL-10 (*r*^2^ = - 0.232, *p*<0.05), KYN (*r*^2^ = 0.556, *p*<0.001), 3-HK (*r*^2^ = 0.297, *p*<0.01), 3-HAA (*r*^2^ = 0.358, *p*<0.001), KYN:TRP (*r*^2^ = - 0.320, *p*<0.01), and 3-HAA:AA (*r*^2^ = 0.236, *p*<0.05) (**Fig 3)**.

## Discussion

The aim of this study was to investigate the effects of acute academic stress on the neuroendocrine, immune mediators and the KP. Academic examination stress has been extensively used to investigate the effects of psychological stress on neuro-endocrino-immunology in healthy young subjects [47]. In this study, we assessed the changes in the levels of cortisol, BDNF, pro- and anti-inflammatory cytokines and several KP metabolites in serum obtained from medical students under the pressure of examination stress compared to a low-stress period. Moreover, as gender is an important determinant of human health, and a clear pattern for the sex-specific stress hormone regulation, prevalence rates of neuro-psychiatric disorders are reported in the literature [48–50]. Hence, the differences in stress reactivity, cytokines and KP metabolites between male and female participants in this study were also analysed.

The DASS-stress scale was designed to assess the levels of chronic nervous arousals [44]. In agreement with previous reports, we found a significant increase in DASS-stress scores among the participants of our study during pre-examination period [30–33]. These participants had behavioural problems such as difficulties in relaxing, feelings of being upset/ agitated, higher irritability, over-reactivity and intolerance to interruption or delay. As the selected participants did not have any psychosocial problems, financial stress, medications and diseases, the cause of their high levels of stress during that examination time-point was considered as the only stress inducer.

Elevated levels of cortisol during the examination period reflect the classic hormonal responses to stress. However, depending on the studies, the cortisol response to academic stress has been found to vary, from no change or even lower levels [36–38] to significantly higher levels [39–41] as we found in this study. The increase in cortisol levels is due to the activation of the HPA axis triggered by examination stress [1–3]. While, significantly higher levels of stress were found during the pre-examination period, an important factor for consideration is the variability in coping abilities and inter-individual variation during high stress periods. In addition, a gender difference was observed in the scores of perceived stress, females exhibited higher stress scores than males under stress. In line with this observation, depression and anxiety disorders are more prevalent in adult women than men [49]. The gonadal hormones are found to have marked effects on physiological stress responses as the hypothalamic pituitary gonadal axis has a close connection with HPA neuronal circuitry [48]. Oestrogen has been shown to buffer the sympathetic and HPA arousal [50] and low serum oestrogen levels are associated with hypoactivation of brain stress responses in women with major depressive disorder [48]. However, no gender difference in cortisol response was noted in this study.

The BDNF is expressed in the hippocampus and cerebral cortex, two brain regions involved in stress regulation [6] as well as in the periphery [51]. BDNF production is decreased during chronic psychological stress [9, 52]. However, to our knowledge, this has never been studied in humans under acute stress conditions. We found that academic stress is associated with a significant increase in BDNF production. This is consistent with the result from a study using a biphasic stress model on sleep, which reported a fast increase in serum BDNF levels as seen in patients with acute stress and, sleep deprivation, but a decrease in levels with chronic stress, sleep disturbance and depression [53]. Previous studies on healthy older adults are limited and inconsistent: no significant changes in serum BDNF levels following a 35-minute sessions [54]; and increased BDNF levels after 5 weeks of cognitive training [55], have been reported. This is also supported by findings with animal models where short-duration stressors of less than 60 minutes triggered an induction of hippocampal BDNF expression [56, 57]. In addition, in animal models with an enriched environment, BDNF and phosphorylated cyclic adenosine monophosphate (cAMP) response element-binding protein (pCREB) improved both hippocampal neurogenesis and cognition [58, 59]. Though the concept of environmental enrichment is far more complex in humans; the pre-examination period could be considered as a state of cognitive stimulation. BDNF mediated neuronal activity and plasticity in response to a cognitive challenge as well as cognitive learning may also explain the high BDNF levels in periods of stress. However, the change in BDNF levels is neither associated with perceived stress nor cortisol levels. Compared to males, female participants showed higher levels of BDNF in stress as well as stress-free periods. This may be due to effect of reproductive hormone, oestrogen, which modulates the expression of neurotrophins [50]. Oestrogen-induced increase in BDNF mRNA and protein levels in cultured hippocampal and prefrontal cortical neurones of rats as well as oestrogen-dependent prefrontal activation in humans have been previously reported [50]. Altogether these points highlight the needs for better characterisation and understanding of the complex and multiple integrated mechanisms involved between perceived stress, cortisol and BDNF production under acute and chronic physiological conditions.

In addition, we observed an alteration of the Th1/Th2 balance between pre- and post-examination periods. A significant decrease of the Th2 anti-inflammatory cytokine (IL-10) production was found in the high-stress period confirming the previous findings from exam stress model of medical students [42, 43]. IL-10 induces a strong humoral response and can suppress the production of the interferon-γ (IFN-γ) and TNF-α by a Th1 clone in a mouse model [60]. However, in this study, even with levels of IL-10 significantly decreased by 1.3-fold during the stress period, there is no significant change in the levels of Th1 pro-inflammatory cytokines (IL-6 and TNF-α). This is likely to be associated with differences between species: mice vs human. Our results support the concept that stress is immunosuppressive [4, 61]. In addition, both interleukins are found to be associated with BDNF in pre- and post-examination periods. This finding is in agreement with the hypothesis of a cross-regulation between BDNF and cytokine production [8] and supported by the several reports: 1) inflammation induced-anxiety and depression with decreased hippocampal expression of BDNF in rats [62]; 2) inflammation-dependent decrease in BDNF in depression [10]; 3) chronic schizophrenia [63]; 4) normalization or up-regulation of BDNF levels in response to antidepressant [64] and 5) antipsychotic treatments [65]. The consensus is that that academic examination induced-stress suppressed the humoral immune response but has minimal effects on the cell-mediated response [4, 61].

Looking at the KP, we did not find any significant differences between the early KP metabolites, TRP and KYN, between the pre- and post-examination periods. The levels of TRP are associated with BDNF and also with downstream KP metabolites, KYN, 3-HK, 3-HAA, KYN:TRP and 3-HAA:AA. This correlates well with the fact that we did not see any changes in the levels of pro-inflammatory cytokines, which are the main activators of IDO-1. However, it was surprising that the high cortisol levels found in the participants did not up-regulate TDO-2 [20]. As mentioned previously, this could be explained by the possible antagonistic role of BDNF on cortisol induced-TDO-2 activation. The central KP metabolite, 3-HK, formed from hydroxylation of KYN by KMO, was increased during post-examination period. The 3-HK is produced by activated monocytic cells, such as microglia and macrophages [24], and activates the neurotoxic branch of KP [16]. The role of 3-HK in neurophysiological process is still not fully understood. A review [66] reported that 3-HK could also have some KP modulatory actions via antioxidant mechanisms as opposed to the previously known pro-oxidant and neurotoxic effects. 3-HK is subsequently catalysed by either KYNU into 3-HAA, or transaminated by KATs into xanthurenic acid (XA) [67] **(Fig 1)**. The significant increase in 3-HK levels during the post-examination period without changes in the levels of its direct catabolite, 3-HAA indicated the possible shift of 3-HK conversion towards XA, and which physiological roles are associated with attentional and cognitive processes by modulating glutamatergic neurotransmission [68]. We also observed that TNF-α can activate KMO directly and independently of IDO-1 activation (unpublished data). 3-HAA is a suppressor of T-cell response [69] and an excitotoxin [70]. 3-HAA is the main precursor for the formation of neurotoxic QUIN [16, 71]. Although significantly lower levels of serum 3-HK at pre- and higher levels of 3-HAA:AA at post-examination period were observed in males, no significant gender difference was found for other kynurenines: TRP, KYN, 3-HK, and KYN:TRP. The similar finding was reported except for plasma TRP which revealed lower total levels in females [72]. The possible significant impact of kynurenines difference in relation to TRP metabolism along KP needs further investigations.

One of the main limitations of this project is that we were not able to quantify the level of XA, QUIN and picolinic acid (PIC) due to limited volume of samples. However, as 3-HK is altered, it is likely that QUIN related neurotoxicity could also prevail under acute stress as previously shown in rodent models [73, 74]. The cortisol awakening time was not determined, this may limit the data on cortisol responses. This notion on the acute stress condition is in accordance with several reports in which chronic stress induced-KP activation, and its involvement in the initiation, development and augmentation of neurodegenerative processes [15, 19, 27–29].

## Conclusion

This study is the first to report the changes in the KP neuroactive metabolites through neuroendocrine-immune interactions in response to acute stress triggered by academic examinations. Increased levels of perceived stress and serum cortisol, reduced levels of anti-inflammatory cytokine, with limited change in peripheral KP neuroactive metabolic profile under the duress of acute stress strongly suggest the main regulatory protagonist could be BDNF. During the pre-examination period, learning induced-cognitive stimulation increases BDNF production, which in turn, attenuates the effects of cortisol and/or the pro-inflammatory cytokines induced by KP activation. Thus, acute stress induced-BDNF may restore the KP metabolic equilibrium and may maintain a basal neuroprotective environment. Future studies with a larger cohort are necessary to provide stronger evidence and to elucidate the mechanisms that control and maintain the KP homeostasis in response to acute stressful conditions in humans.

## Supporting information

S1 File Standard curves for calculation of kynurenine metabolite concentrations. (PDF)

S2 File Project data sheet. (PDF)
