# Supplementary material for: Effects of stress associated with academic examination on the kynurenine pathway profile in healthy students
Source: PLoS One. 2021 Jun 3;16(6):e0252668. doi: 10.1371/journal.pone.0252668 (PMC8174692; doi:10.1371/journal.pone.0252668)
Supplement: S1 File — (PDF) [file pone.0252668.s001.pdf]

S1: Standard Curves for Calculation of kynurenine metabolite concentrations

um **kyn**	um **tryp**		**kyn** RT	dad area	**tryp** RT	ex280 area
10	50	KT4	3.202	64.7	7.565	4059.1
5	10	kt3	3.191	30.4	7.53	818.1
2	5	kt2	3.144	8.4	7.446	411.3
1	2	kt1	3.007	4.4	7.134	166.9

a)

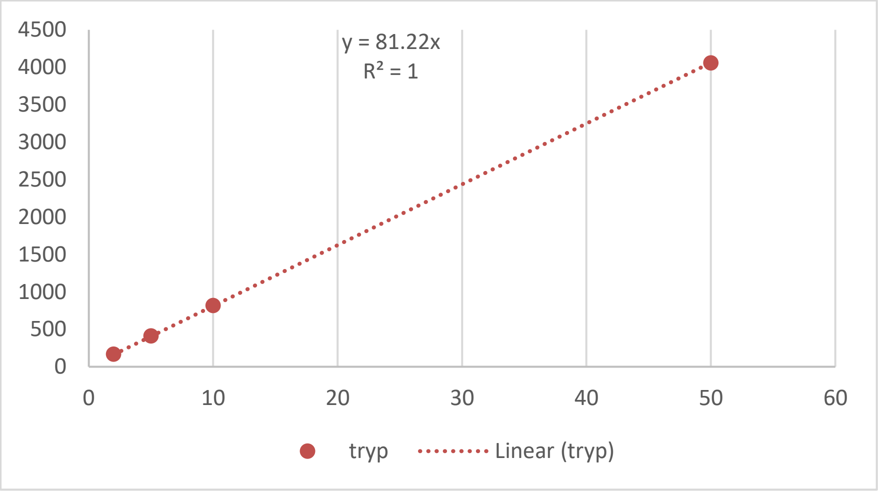

b)

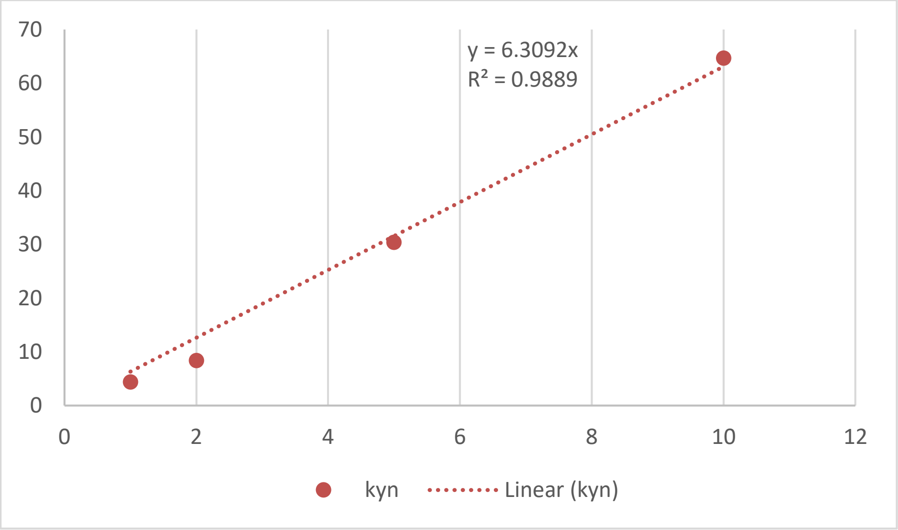

Figure S1: Standard curves for determination of serum (a) TRP and (b) KYN concentration

nm	3HK RT	dad area	3HAA RT	ex320 area	AA RT	ex320 area
100	1.241	0.45	3.18	16.8	9.339	17.4
50	1.242	0.25	3.178	8.5	9.34	8.4
20	1.241	0.1	3.18	3.1	9.34	3.5
5	1.236	0.05	3.18	0.8	9.331	0.96

a)

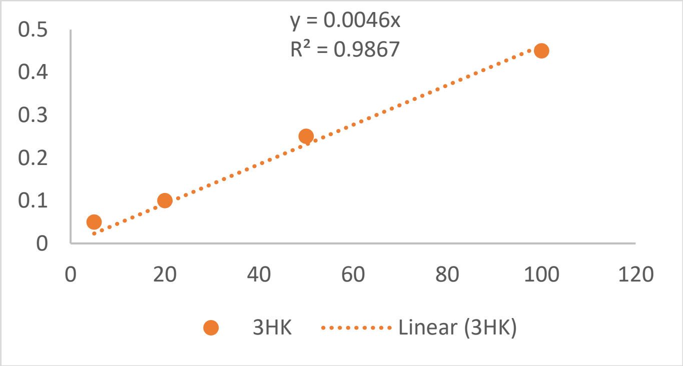

b)

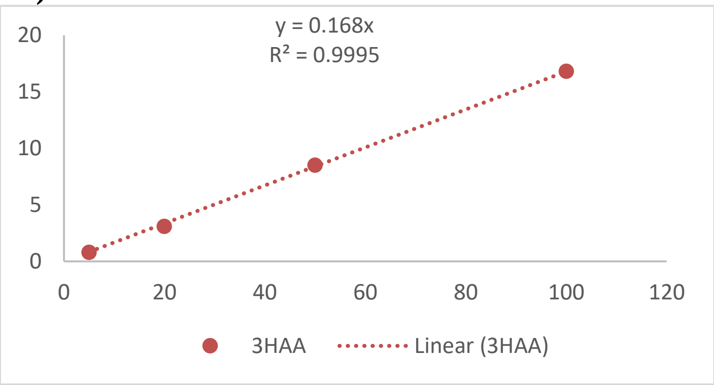

c)

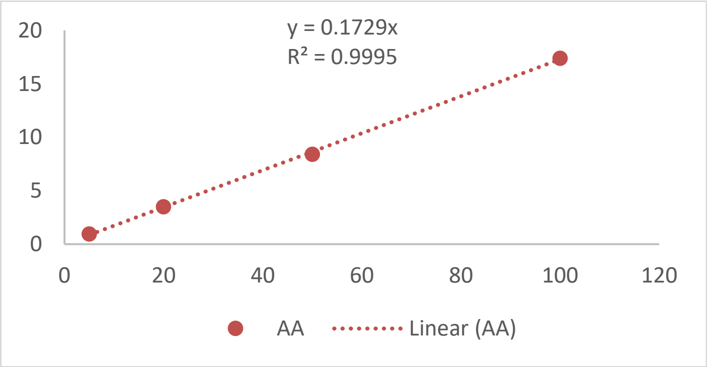

Figure S2: Standard curves for determination of serum (a) 3-HK and (b) 3-HAA and (c) AA concentration
