# Supplementary material for: Effects of stress associated with academic examination on the kynurenine pathway profile in healthy students
Source: PLoS One. 2021 Jun 3;16(6):e0252668. doi: 10.1371/journal.pone.0252668 (PMC8174692; doi:10.1371/journal.pone.0252668)
Supplement: S2 File — (PDF) [file pone.0252668.s002.pdf]

Data for project "Effects of stress associated with academic examination on the kynurenine pathway profile in healthy students"

coding	stats_id	prepost	age	gender	ehnics	bldgroup	letal status	ding course	ending year	ceproblem	smoking	drinking	medical ill	ntal illness	bmi	p_stress	cortisol (nmol/L)	bdnf (pg/ml)	il-10 (pg/ml)	il-6 (pg/ml)	tnf alpha	3-hk (nM)	kyn (uM)	trp (uM)	3-haa (nM)	aa (nM)	kyn:trp *1000	3-haa:aa
101	1	1	20	1	2	3	1	1	1	0	0	1	0	0	30.3	12.00	510.00	16.49	0.80	0.24	1.18	43.48	1.20	42.60	25.60	32.39	28.27	0.79
	1	2	20	1	2	3	1	1	1	0	0	1	0	0		2.00	522.00	2.07	1.43	0.01	1.35	56.52	1.14	38.04	19.64	40.49	30.00	0.49
103	2	1	20	2	2	3	1	1	1	0	0	0	0	0	19.1	16.00	556.00	25.03	1.04	0.24	1.01	34.78	0.89	32.50	13.10	28.34	27.31	0.46
	2	2	20	2	2	3	1	1	1	0	0	0	0	0		0.00	459.00	104.08	0.84	0.01	1.94	47.83	0.95	34.60	10.71	30.08	27.48	0.36
104	3	1	20	1	3	3	1	1	1	0	1	1	0	0	28.5	2.00	608.00	116.60	1.25	0.24	1.52	30.43	0.67	22.62	11.31	39.91	29.43	0.28
	3	2	20	1	3	3	1	1	1	0	1	1	0	0		1.00	526.00	197.94	1.30	0.01	1.65	45.65	1.17	24.53	17.86	34.12	47.81	0.52
105	4	1	20	2	1	1	1	1	1	0	0	0	0	0	18.7	11.00	496.00	188.58	1.36	0.01	1.94	43.48	1.39	29.09	18.45	21.98	47.96	0.84
	4	2	20	2	1	1	1	1	1	0	0	0	0	0		6.00	457.00	101.80	0.84	0.01	1.94	54.35	1.01	31.81	18.45	27.76	31.89	0.66
106	5	1	20	2	1	3	1	1	1	0	0	0	0	0	18.7	18.00	510.00	6.41	0.63	0.01	1.65	60.87	1.17	26.19	18.45	30.65	44.79	0.60
	5	2	20	2	1	3	1	1	1	0	0	0	0	0		12.00	303.00	27.81	0.90	0.02	1.35	60.87	1.52	26.44	23.21	28.34	57.55	0.82
107	6	1	20	1	2	2	1	1	1	0	1	0	0	0	20.2	15.00	310.00	55.49	0.68	0.24	0.49	34.78	0.86	30.17	22.02	58.42	28.37	0.38
	6	2	20	1	2	2	1	1	1	0	1	0	0	0		10.00	363.00	5.96	1.82	0.02	1.65	50.00	0.92	32.71	21.43	60.15	28.10	0.36
108	7	1	20	1	2	1	1	1	1	0	0	0	0	0	21.8	14.00	509.00	181.96	1.11	0.24	2.21	89.13	1.44	40.58	20.24	26.60	35.54	0.76
	7	2	20	1	2	1	1	1	1	0	0	0	0	0		11.00	439.00	67.78	1.43	0.02	2.24	104.35	1.33	36.29	20.24	40.49	36.69	0.50
109	8	1	20	1	2	2	1	1	1	0	0	1	0	0	19.6	8.00	448.00	105.85	1.60	0.25	2.05	34.78	1.60	34.62	25.00	40.49	46.24	0.62
	8	2	20	1	2	2	1	1	1	0	0	1	0	0		8.00	422.00	30.43	1.43	8.60	2.54	39.13	1.65	36.09	31.55	42.80	45.67	0.74
110	9	1	20	1	2	3	1	1	1	0	0	0	0	0	20.4	10.00	519.00	85.45	0.95	0.24	1.87	58.70	1.57	51.31	35.12	34.12	30.58	1.03
	9	2	20	1	2	3	1	1	1	0	0	0	0	0		0.00	340.00	29.76	1.17	0.01	1.36	38.64	0.83	30.30	23.02	45.10	27.39	0.51
111	10	1	19	1	2	1	1	1	1	0	1	1	0	0	23.3	14.00	567.00	35.14	1.60	0.24	0.84	30.43	1.00	24.50	14.88	23.71	40.76	0.63
	10	2	19	1	2	1	1	1	1	0	1	1	0	0		20.00	536.00	18.29	1.86	1.03	1.36	40.91	1.03	27.59	14.76	37.37	37.32	0.39
112	11	1	20	1	2	1	1	1	1	0	0	0	0	0	24.2	18.00	399.00	250.37	0.04	14.75	1.18	41.30	1.06	37.11	25.00	34.70	28.62	0.72
	11	2	20	1	2	1	1	1	1	0	0	0	0	0		8.00	518.00	181.71	0.56	18.45	1.80	59.09	1.06	37.98	24.20	45.75	27.92	0.53
113	12	1	20	1	2	1	1	1	1	0	0	0	0	0	20.5	13.00	287.00	57.53	1.11	0.24	1.53	43.48	1.52	38.28	37.50	45.11	39.75	0.83
	12	2	20	1	2	1	1	1	1	0	0	0	0	0		13.00	420.00	8.93	1.17	0.01	1.06	61.36	1.23	36.06	26.56	34.15	34.10	0.78
114	13	1	20	1	2	1	1	1	1	0	0	1	0	0	21.3	6.00	374.00	42.03	1.39	0.24	2.90	36.96	1.20	35.48	16.67	39.33	33.95	0.42
	13	2	20	1	2	1	1	1	1	0	0	1	0	0		6.00	373.00	21.40	1.04	0.01	2.54	68.18	1.74	52.53	40.14	33.51	33.06	1.20
115	14	1	20	2	2	1	1	1	1	0	0	0	0	0	21.0	17.00	572.00	401.57	0.60	0.24	1.52	32.61	0.97	27.58	13.69	46.27	35.05	0.30
	14	2	20	2	2	1	1	1	1	0	0	0	0	0		5.00	384.00	98.66	1.11	0.01	1.36	56.82	1.35	42.56	25.38	52.19	31.78	0.49
116	15	1	20	1	2	3	1	1	1	0	1	0	0	0	21.8	7.00	448.00	44.67	1.50	0.24	0.48	36.96	1.14	27.40	16.07	83.29	41.65	0.19
	15	2	20	1	2	3	1	1	1	0	1	0	0	0		13.00	498.00	12.45	1.82	0.01	1.65	68.18	1.58	47.28	37.78	37.37	33.48	1.01
117	16	1	20	1	2	3	1	1	1	0	0	0	0	0	22.7	10.00	510.00	94.37	0.25	0.24	1.52	17.39	0.92	25.30	11.90	86.18	36.33	0.14
	16	2	20	1	2	3	1	1	1	0	0	0	0	0		0.00	540.00	41.45	0.67	0.02	1.36	56.82	1.26	42.59	21.84	30.28	29.59	0.72
118	17	1	19	1	2	3	1	1	1	0	0	0	0	0	29.9	22.00	243.00	295.83	0.22	0.24	1.52	50.00	1.36	39.08	34.52	34.70	34.88	0.99
	17	2	19	1	2	3	1	1	1	0	0	0	0	0		0.00	455.00	27.62	0.84	0.01	1.65	54.55	1.43	43.91	25.38	35.44	32.55	0.72
119	18	1	21	1	2	3	1	1	1	0	0	1	0	0	23.6	7.00	200.00	129.78	0.38	0.24	0.84	34.78	1.11	38.44	23.21	42.80	28.86	0.54
	18	2	21	1	2	3	1	1	1	0	0	1	0	0		7.00	435.00	17.16	0.37	0.02	1.65	59.09	1.40	39.13	34.83	71.52	35.74	0.49
120	19	1	21	1	2	3	1	1	1	0	0	0	0	0	22.5	9.00	477.00	131.78	0.75	0.24	0.84	34.78	1.17	29.77	19.64	68.25	39.40	0.29
	19	2	21	1	2	3	1	1	1	0	0	0	0	0		3.00	537.00	23.83	0.77	0.01	1.36	31.82	0.92	27.38	13.58	74.10	33.68	0.18
121	20	1	20	1	2	2	1	1	1	0	0	1	0	0	18.9	12.00	570.00	15.85	0.17	0.24	1.53	30.43	1.11	34.93	13.69	45.69	31.77	0.30
	20	2	20	1	2	2	1	1	1	0	0	1	0	0		10.00	643.00	24.20	1.23	0.01	1.36	54.55	1.17	37.88	15.35	44.46	30.84	0.35
122	21	1	20	1	2	2	1	1	1	0	0	0	0	0	24.2	7.00	524.00	21.99	1.57	0.24	1.52	71.74	1.46	41.15	20.83	245.81	35.44	0.08
	21	2	20	1	2	2	1	1	1	0	0	0	0	0		4.00	456.00	14.31	1.30	0.01	2.54	84.09	1.09	22.68	24.20	23.84	48.10	1.02
123	22	1	20	1	2	4	1	1	1	0	0	0	0	0	21.4	15.00	543.00	29.20	1.60	0.24	0.49	50.00	1.35	30.41	14.88	42.80	44.31	0.35
	22	2	20	1	2	4	1	1	1	0	0	0	0	0		21.00	511.00	2.86	0.23	0.01	1.06	84.09	1.60	47.16	21.84	34.79	33.89	0.63
124	23	1	19	1	2	3	1	1	1	0	1	1	0	0	24.2	6.00	462.00	27.77	0.46	0.24	0.84	36.96	1.39	42.18	22.02	42.80	33.07	0.51
	23	2	19	1	2	3	1	1	1	0	1	1	0	0		2.00	433.00	53.33	0.74	0.01	1.65	56.82	1.40	39.70	36.01	72.16	35.22	0.50
125	24	1	21	2	2	3	1	1	1	0	0	0	0	0	21.6	9.00	384.00	43.55	0.97	0.24	0.48	56.52	1.30	35.54	32.14	47.43	36.57	0.68
	24	2	21	2	2	3	1	1	1	0	0	0	0	0		5.00	261.00	6.94	1.17	0.01	1.35	63.64	1.12	31.86	27.74	42.53	35.21	0.65
126	25	1	20	1	1	2	1	1	1	0	0	0	0	0	21.4	6.00	453.00	78.92	2.13	0.24	0.84	80.43	1.82	33.87	23.21	78.08	53.82	0.30
	25	2	20	1	1	2	1	1	1	0	0	0	0	0		3.00	406.00	19.81	3.20	0.01	1.65	79.55	2.04	37.31	18.89	72.16	54.79	0.26
127	26	1	20	1	1	1	1	1	1	0	0	0	0	0	22.4	6.00	513.00	73.26	0.83	0.24	1.70	30.43	0.86	21.17	25.00	43.38	40.43	0.58
	26	2	20	1	1	1	1	1	1	0	0	0	0	0		7.00	290.00	44.33	0.88	0.01	1.50	70.45	1.66	29.81	52.54	79.90	55.67	0.66
128	27	1	20	2	1	2	1	1	1	0	0	0	0	0	19.6	10.00	404.00	25.47	0.97	0.24	0.66	26.09	0.74	19.93	11.90	45.69	37.38	0.26
	27	2	20	2	1	2	1	1	1	0	0	0	0	0		0.00	288.00	19.61	0.84	0.01	1.06	77.27	1.43	36.97	28.93	42.53	38.66	0.68
129	28	1	20	2	1	2	1	1	1	0	0	0	0	0	21.1	15.00	499.00	44.85	0.25	0.24	1.18	80.43	1.49	36.10	33.93	46.85	41.27	0.72
	28	2	20	2	1	2	1	1	1	0	0	0	0	0		7.00	576.00	80.19	0.57	0.02	1.51	72.73	1.43	37.09	26.56	56.06	38.54	0.47
132	29	1	20	2	1	1	1	1	1	0	0	0	0	0	17.7	12.00	354.00	201.14	0.30	0.24	1.18	54.35	1.65	41.95	20.83	49.16	39.29	0.42
	29	2	20	2	1	1	1	1	1	0	0	0	0	0		5.00	397.00	982.16	0.84	0.01	2.68	59.09	1.17	35.17	14.76	45.75	33.21	0.32
134	30	1	20	2	1	3	1	1	1	0	0	0	0	0	19.4	20.00	512.00	108.99	0.43	0.24	0.66	34.78	0.74	19.38	13.10	21.98	38.43	0.60
	30	2	20	2	1	3	1	1	1	0	0	0	0	0		24.00	488.00	66.33	1.04	3.48	2.39	17.27	0.43	15.67	7.08	103.09	27.46	0.07
135	31	1	20	2	1	1	1	1	1	0	0	0	0	0	17.7	14.00	354.00	151.32	0.52	0.24	1.18	82.61	1.66	40.94	24.40	41.06	40.65	0.59
	31	2	20	2	1	1	1	1	1	0	0	0	0	0		3.00	448.00	22.04	1.63	0.02	1.36	65.91	1.18	33.26	19.48	131.44	35.58	0.15
136	32	1	20	2	1	2	1	1	1	0	0	0	0	0	23.4	14.00	529.00	19.88	0.71	0.24	1.01	30.43	0.54	16.06	10.12	25.45	33.56	0.40

	32	2	20	2	1	2	1	1	1	0	0	0	0	0		1.00	273.00	41.72	0.98	0.01	1.65	54.55	0.74	23.43	14.76	30.93	31.48	0.48
137	33	1	21	2	1	2	1	1	1	0	0	0	0	0	21.8	21.00	369.00	10.55	0.26	0.24	1.18	34.78	0.71	24.14	17.26	63.62	29.55	0.27
	33	2	21	2	1	2	1	1	1	0	0	0	0	0		4.00	145.00	78.27	0.44	0.01	1.06	65.91	0.89	30.95	28.93	35.44	28.80	0.82
139	34	1	20	2	1	2	1	1	1	0	0	0	0	0	28.6	20.00	620.00	4.53	0.38	0.24	1.35	56.52	1.74	36.26	26.79	79.81	48.09	0.34
	34	2	20	2	1	2	1	1	1	0	0	0	0	0		20.00	520.00	733.26	0.54	0.01	2.24	61.36	1.29	28.46	20.66	82.47	45.36	0.25
141	35	1	20	1	1	3	1	1	1	0	0	0	0	0	23.0	6.00	268.00	13.85	0.90	0.24	0.84	41.30	0.90	28.27	22.02	34.70	31.96	0.63
	35	2	20	1	1	3	1	1	1	0	0	0	0	0		5.00	577.00	67.11	169.62	20.67	3.99	47.73	1.43	18.93	20.07	119.20	75.51	0.17
142	36	1	20	1	1	3	1	1	1	0	0	0	0	0	23.4	9.00	586.00	189.51	0.59	0.24	0.84	23.91	1.20	26.30	20.83	74.61	45.80	0.28
	36	2	20	1	1	3	1	1	1	0	0	0	0	0		6.00	504.00	1840.60	0.28	0.20	0.58	75.00	0.78	21.67	17.12	43.17	36.16	0.40
144	37	1	20	2	1	3	1	1	1	0	0	0	0	0	16.7	18.00	145.00	75.42	2.59	0.24	1.18	56.52	1.20	40.83	35.12	48.58	29.50	0.72
	37	2	20	2	1	3	1	1	1	0	0	0	0	0		2.00	408.00	63.04	2.03	65.58	34.52	68.18	1.28	38.66	20.66	102.45	32.99	0.20
145	38	1	20	1	3	4	1	1	1	0	1	1	0	0	34.1	9.00	233.00	30.29	0.17	0.24	0.83	43.48	1.30	34.76	13.10	31.81	37.39	0.41
	38	2	20	1	3	4	1	1	1	0	1	1	0	0		5.00	441.00	213.49	0.37	0.61	1.65	43.18	0.92	26.89	23.02	49.61	34.29	0.46
146	39	1	19	1	3	2	1	1	1	0	1	1	0	0	29.1	26.00	445.00	130.27	1.95	1.07	1.01	52.17	1.82	42.79	32.74	48.58	42.60	0.67
	39	2	19	1	1	2	1	1	1	0	1	1	0	0		9.00	359.00	84.23	3.70	2.08	1.65	54.55	1.35	34.24	28.93	47.04	39.50	0.61
147	40	1	21	2	1	3	1	1	1	0	0	0	0	0	19.6	13.00	309.00	12.12	1.11	0.24	0.84	50.00	1.78	32.61	25.00	57.26	54.43	0.44
	40	2	21	2	1	3	1	1	1	0	0	0	0	0		11.00	201.00	96.18	1.43	2.39	1.65	54.55	1.52	33.89	20.07	37.37	44.90	0.54
148	41	1	20	1	1	3	1	1	1	0	0	0	0	0	20.3	17.00	461.00	27.97	1.24	2.76	1.65	36.96	1.32	33.41	27.38	49.16	39.37	0.56
	41	2	20	1	1	3	1	1	1	0	0	0	0	0		5.00	358.00	217.77	1.37	1.03	1.65	56.82	1.34	35.97	43.68	89.56	37.17	0.49
301	42	1	20	2	1	3	1	1	1	0	0	0	0	0	18.2	18.00	440.00	919.76	1.60	0.24	2.56	70.45	1.51	35.35	18.89	65.08	42.60	0.29
	42	2	20	2	1	3	1	1	1	0	0	0	0	0		18.00	422.00	838.60	0.23	0.72	1.00	129.55	0.75	17.14	14.76	56.06	43.93	0.26
302	43	1	20	2	1	3	1	1	1	0	0	0	0	0	26.1	9.00	386.00	1001.23	0.11	0.72	1.00	61.36	0.98	37.88	23.02	50.90	25.97	0.45
	43	2	20	2	1	3	1	1	1	0	0	0	0	0		12.00	412.00	775.60	0.36	1.02	0.58	52.27	1.26	29.35	32.47	41.88	42.94	0.78
303	44	1	20	2	1	3	1	1	1	0	0	0	0	0	22.9	18.00	517.00	1190.55	0.88	0.72	1.00	63.64	1.64	42.23	32.47	116.62	38.94	0.28
	44	2	20	2	1	3	1	1	1	0	0	0	0	0		15.00	468.00	1329.30	0.36	0.72	0.77	68.18	1.43	30.58	34.24	83.76	46.74	0.41
304	45	1	20	2	1	1	1	1	1	0	0	0	0	0	17.7	16.00	390.00	988.11	0.06	0.72	1.00	59.09	1.34	41.93	24.79	56.06	31.89	0.44
	45	2	20	2	1	1	1	1	1	0	0	0	0	0		14.00	345.00	516.01	0.06	0.72	0.10	50.00	1.14	41.64	18.89	41.88	27.31	0.45
305	46	1	20	2	1	3	1	1	1	0	0	0	0	0	20.9	9.00	492.00	1592.15	0.11	0.72	0.32	61.36	1.28	47.78	40.14	74.10	26.70	0.54
	46	2	20	2	1	3	1	1	1	0	0	0	0	0		1.00	375.00	1844.05	0.06	0.72	1.21	70.45	1.37	41.94	34.83	40.59	32.62	0.86
306	47	1	19	2	1	3	1	1	1	0	0	0	0	0	18.1	13.00	602.00	1603.25	0.06	0.72	0.54	54.55	0.86	3.14	13.58	34.15	274.06	0.40
	47	2	19	2	1	3	1	1	1	0	0	0	0	0		11.00	556.00	2304.99	0.06	1.31	0.78	63.64	1.00	37.22	17.71	36.08	26.84	0.49
307	48	1	20	2	2	2	1	1	1	0	0	0	0	0	20.6	11.00	574.00	1519.67	0.43	0.72	1.00	40.91	0.75	2.87	28.93	36.08	262.66	0.80
	48	2	20	2	2	2	1	1	1	0	0	0	0	0		1.00	350.00	1778.71	3.31	0.72	0.77	52.27	1.40	27.25	36.60	38.02	51.33	0.96
308	49	1	20	2	2	3	1	1	1	0	0	0	0	0	20.9	12.00	432.00	815.07	1.70	0.72	0.32	52.27	0.85	27.42	27.74	34.79	30.83	0.80
	49	2	20	2	2	3	1	1	1	0	0	0	0	0		13.00	453.00	675.47	1.06	0.20	0.40	63.64	2.70	56.29	27.74	276.42	48.05	0.10
309	50	1	21	2	2	3	1	1	1	0	0	0	0	0	20.4	25.00	524.00	917.09	1.06	0.72	0.54	56.82	0.97	32.48	17.12	44.46	29.81	0.39
	50	2	21	2	2	3	1	1	1	0	0	0	0	0		5.00	344.00	438.74	3.33	0.20	0.58	70.45	1.55	27.26	25.38	47.04	56.95	0.54
310	51	1	20	2	2	3	1	1	1	0	0	0	0	0	21.2	9.00	429.00	1824.85	0.18	0.72	0.54	40.91	1.14	39.26	15.94	240.34	28.97	0.07
	51	2	20	2	2	3	1	1	1	0	0	0	0	0		4.00	367.00	1994.43	0.12	0.20	0.10	68.18	1.23	33.18	21.25	201.03	37.05	0.11
311	52	1	20	1	2	3	1	1	1	0	0	0	0	0	21.2	7.00	420.00	439.37	0.42	0.72	1.00	65.91	1.38	39.28	31.29	38.66	35.22	0.81
	52	2	20	1	2	3	1	1	1	0	0	0	0	0		5.00	377.00	529.64	1.76	0.74	0.54	59.09	1.54	28.19	27.74	37.37	54.51	0.74
312	53	1	20	2	1	1	1	1	1	0	0	0	0	0	20.4	13.00	612.00	2363.66	0.24	0.72	1.89	72.73	1.21	50.55	42.50	47.68	24.02	0.89
	53	2	20	2	1	1	1	1	1	0	0	0	0	0		12.00	624.00	2688.80	0.19	0.20	1.76	90.91	1.18	32.66	33.65	38.66	36.23	0.87
313	54	1	20	2	1	1	1	1	1	0	0	0	0	0	20.8	16.00	604.00	1056.29	0.85	0.72	0.32	50.00	0.86	27.63	22.43	53.48	31.15	0.42
	54	2	20	2	1	1	1	1	1	0	0	0	0	0		17.00	588.00	899.30	0.72	0.20	0.58	63.64	1.32	35.27	18.89	21.91	37.48	0.86
314	55	1	20	1	1	2	1	1	1	0	0	0	0	0	22.3	25.00	567.00	498.18	0.68	0.72	0.27	43.18	1.23	35.98	20.07	39.30	34.17	0.51
	55	2	20	1	1	2	1	1	1	0	0	0	0	0		22.00	532.00	361.20	0.56	0.72	0.32	61.36	1.49	35.53	21.84	39.95	41.95	0.55
315	56	1	20	1	2	1	1	1	1	0	0	0	0	0	24.9	9.00	512.00	1427.08	1.03	0.72	1.46	52.27	1.03	43.40	23.02	44.46	23.73	0.52
	56	2	20	1	2	1	1	1	1	0	0	0	0	0		4.00	398.00	799.00	0.71	0.20	1.23	31.82	0.75	29.67	10.63	62.50	25.38	0.17
316	57	1	20	2	2	2	1	1	1	0	0	0	0	0	17.4	11.00	457.00	3194.68	0.29	0.72	1.00	59.09	1.18	42.81	25.38	99.23	27.64	0.26
	57	2	20	2	2	2	1	1	1	0	0	0	0	0		5.00	365.00	2246.48	0.07	0.20	0.93	75.00	1.46	57.21	28.93	36.73	25.52	0.79
317	58	1	20	2	2	3	1	1	1	0	0	0	0	0	18.8	13.00	488.00	2007.86	0.94	0.72	0.54	81.82	1.24	42.64	41.91	53.48	29.20	0.78
	58	2	20	2	2	3	1	1	1	0	0	0	0	0		7.00	373.00	1581.42	1.38	0.20	0.58	56.82	1.18	29.02	32.47	45.10	40.78	0.72
318	59	1	20	1	1	3	1	1	1	0	0	0	0	0	27.8	9.00	412.00	542.55	2.07	0.72	0.32	50.00	1.77	28.18	21.25	51.55	62.71	0.41
	59	2	20	1	1	3	1	1	1	0	0	0	0	0		7.00	377.00	1029.81	1.42	0.20	1.41	61.36	1.41	33.15	27.15	28.35	42.65	0.96
319	60	1	20	1	2	3	1	1	1	0	0	0	0	0	24.9	20.00	466.00	745.64	0.31	0.72	0.32	38.64	1.12	42.11	17.71	57.99	26.64	0.31
	60	2	20	1	2	3	1	1	1	0	0	0	0	0		7.00	282.00	108.98	0.51	0.20	0.10	63.64	1.97	41.49	37.19	109.54	47.41	0.34
320	61	1	20	1	1	3	1	1	1	0	0	0	0	0	18.1	6.00	378.00	643.10	0.42	0.72	1.00	70.45	1.60	42.91	50.77	35.44	37.24	1.43
	61	2	20	1	1	3	1	1	1	0	0	0	0	0		3.00	356.00	917.45	0.36	0.72	0.10	61.36	1.72	44.60	24.20	25.13	38.60	0.96
350	62	1	20	1	2	2	1	1	1	0	0	0	0	0	21.3	2.00	389.00	2876.16	1.64	7.89	9.73	63.64	1.17	38.19	26.56	28.35	30.58	0.94
	62	2	20	1	2	2	1	1	1	0	0	0	0	0		0.00	347.00	1323.92	0.15	0.20	0.40	50.00	0.94	30.80	21.25	27.71	30.43	0.77
351	63	1	20	2	2	3	1	1	1	0	0	0	0	0	20.4	15.00	567.00	639.46	1.59	0.73	0.54	63.64	1.06	45.79	38.37	52.84	23.16	0.73
	63	2	20	2	2	3	1	1	1	0	0	0	0	0		9.00	474.00	849.17	2.20	0.20	0.10	38.64	0.86	27.52	13.58	74.10	31.28	0.18
352	64	1	20	2	2	4	1	1	1	0	0	0	0	0	18.4	10.00	444.00	808.05	0.42	0.72	1.46	81.82	1.38	49.48	28.34	38.02	27.96	0.75
	64	2	20	2	2	4	1	1	1	0	0	0	0	0		10.00	312.00	413.06	0.20	0.20	1.06	45.45	0.94	29.29	16.53	45.10	32.01	0.37
353	65	1	20	1	2	2	1	1	1	0	1	0	0	0	22.1	12.00	432.00	1491.46	0.75	6.50	0.54	61.36	1.40	49.95	16.53	48.32	28.00	0.34
	65	2	20	1	2	2	1	1	1	0	1	0	0	0		15.00	418.00	1155.15	0.51	0.20	0.88	45.45	1.34	46.12	22.43	40.59	28.99	0.55
354	66	1	21	1	2	3	1	1	1	0	0	1	0	0	21.3	14.00	514.00	1381.43	0.11	0.72	1.43	97.73	1.74	70.80	54.31	41.88	24.53	1.30

	66	2	21	1	2	3	1	1	1	0	0	1	0	0		0.00	396.00	922.75	0.36	0.20	0.10	104.55	1.54	45.38	41.32	27.71	33.87	1.49
355	67	1	20	2	1	2	1	1	1	0	0	0	0	0	20.1	7.00	572.00	2087.54	1.06	0.72	1.43	90.91	1.15	40.98	41.32	39.95	28.13	1.03
	67	2	20	2	1	2	1	1	1	0	0	0	0	0		0.00	438.00	1497.07	3.53	0.20	0.58	63.64	1.60	34.02	20.07	43.17	46.99	0.46
356	68	1	20	1	1	4	1	1	1	0	0	0	0	0	23.3	6.00	566.00	1677.95	0.62	2.61	3.16	63.64	1.43	36.06	27.74	57.99	39.63	0.48
	68	2	20	1	1	4	1	1	1	0	0	0	0	0		2.00	475.00	1150.34	0.72	0.20	1.41	61.36	1.58	43.07	23.02	54.77	36.76	0.42
357	69	1	20	2	1	3	1	1	1	0	0	0	0	0	21.4	20.00	602.00	345.90	2.51	7.20	5.08	54.55	1.29	27.42	18.89	34.15	47.08	0.55
	69	2	20	2	1	3	1	1	1	0	0	0	0	0		20.00	551.00	1235.82	1.26	0.30	0.40	61.36	1.34	29.47	12.40	26.42	45.37	0.47
358	70	1	20	2	2	3	1	1	1	0	0	0	0	0	19.3	16.00	473.00	1225.93	1.21	0.72	7.47	65.91	1.17	32.19	36.60	77.32	36.29	0.47
	70	2	20	2	2	3	1	1	1	0	0	0	0	0		15.00	434.00	715.73	1.19	0.20	0.10	47.73	1.12	33.12	15.94	66.37	33.87	0.24
359	71	1	20	1	2	1	1	1	1	0	0	1	0	0	20.1	4.00	334.00	1423.46	0.21	0.72	4.57	50.00	0.89	38.69	11.22	35.44	23.04	0.32
	71	2	20	1	2	1	1	1	1	0	0	1	0	0		2.00	298.00	971.09	0.44	0.20	0.58	59.09	1.17	48.32	12.99	28.99	24.17	0.45
360	72	1	20	1	1	1	1	1	1	0	0	0	0	0	21.6	12.00	550.00	1118.70	0.94	0.72	1.00	56.82	1.17	38.28	26.56	62.50	30.51	0.43
	72	2	20	1	1	1	1	1	1	0	0	0	0	0		14.00	535.00	549.94	0.86	0.20	0.58	59.09	1.28	37.55	24.79	47.04	33.97	0.53
361	73	1	20	1	1	2	1	1	1	0	0	0	0	0	19.3	18.00	465.00	344.97	0.11	0.72	0.32	59.09	1.46	37.58	20.66	43.81	38.85	0.47
	73	2	20	1	1	2	1	1	1	0	0	0	0	0		21.00	481.00	585.73	1.06	0.20	0.10	47.73	1.35	39.49	21.84	51.55	34.24	0.42
362	74	1	20	1	1	3	1	1	1	0	0	0	0	0	22.6	12.00	374.00	392.09	0.19	0.72	0.54	45.45	1.35	44.32	17.12	52.19	30.52	0.33
	74	2	20	1	1	3	1	1	1	0	0	0	0	0		9.00	355.00	699.82	0.36	0.20	0.58	43.18	1.18	38.03	17.12	45.75	31.11	0.37
501	75	1	20	1	1	3	1	1	1	0	0	0	0	0	16.0	3.00	423.00	1063.64	0.54	0.20	0.58	90.91	1.83	51.95	25.38	47.68	35.21	0.53
	75	2	20	1	1	3	1	1	1	0	0	0	0	0		0.00	397.00	1272.92	0.40	1.24	1.36	70.45	1.29	38.54	18.30	37.37	33.50	0.49
502	76	1	20	1	1	3	1	1	1	0	0	0	0	0	23.5	7.00	511.00	1622.91	1.12	0.20	0.10	75.00	1.71	50.36	30.70	39.95	33.87	0.77
	76	2	20	1	1	3	1	1	1	0	0	0	0	0		7.00	438.00	884.75	0.50	1.24	2.78	65.91	1.46	44.41	24.20	48.32	32.87	0.50
504	77	1	20	2	2	3	1	1	1	0	0	0	0	0	19.1	6.00	546.00	1033.76	0.01	0.20	0.40	56.82	1.49	54.74	26.56	36.08	27.23	0.74
	77	2	20	2	2	3	1	1	1	0	0	0	0	0		3.00	491.00	168.11	0.37	1.24	0.58	34.09	0.83	27.06	19.95	37.37	30.67	0.10
505	78	1	19	2	1	3	1	1	1	0	0	0	0	0	21.5	10.00	438.00	926.79	0.07	0.20	0.93	63.64	1.46	45.88	14.17	47.68	31.82	0.30
	78	2	19	2	1	3	1	1	1	0	0	0	0	0		12.00	409.00	88.44	0.46	1.24	0.38	70.45	1.66	53.74	20.07	56.06	30.88	0.36
506	79	1	19	2	1	1	1	1	1	0	0	0	0	0	20.2	8.00	463.00	1707.08	0.51	0.20	1.76	84.09	1.97	53.80	12.99	54.77	36.56	0.24
	79	2	19	2	1	1	1	1	1	0	0	0	0	0		11.00	395.00	49.60	0.22	1.24	0.16	50.00	1.15	29.98	11.81	77.96	38.44	0.15
507	80	1	20	1	2	2	1	1	1	0	0	1	0	0	27.6	6.00	478.00	1529.07	2.71	0.20	0.58	75.00	1.91	54.31	38.37	37.37	35.09	1.03
	80	2	20	1	2	2	1	1	1	0	0	1	0	0		2.00	394.00	3.69	0.03	1.24	0.60	84.09	1.91	55.77	51.36	43.81	34.17	1.17
508	81	1	19	2	1	2	1	1	1	0	0	0	0	0	21.9	15.00	594.00	3339.24	1.52	0.20	2.81	63.64	1.43	39.46	22.43	110.82	36.22	0.20
	81	2	19	2	1	2	1	1	1	0	0	0	0	0		9.00	488.00	627.61	0.63	3.88	0.77	52.27	1.34	41.85	10.63	65.08	31.95	0.16
509	82	1	20	2	1	2	1	1	1	0	0	0	0	0	21.5	11.00	514.00	656.39	0.51	0.20	0.58	63.64	1.74	48.67	29.52	53.48	35.68	0.55
	82	2	20	2	1	2	1	1	1	0	0	0	0	0		6.00	493.00	1333.67	0.92	1.24	1.36	56.82	1.24	38.88	20.66	49.61	32.02	0.42
510	83	1	20	2	1	1	1	1	1	0	0	0	0	0	19.5	10.00	554.00	1395.83	0.76	0.20	1.06	50.00	1.37	37.51	25.97	212.63	36.46	0.12
	83	2	20	2	1	1	1	1	1	0	0	0	0	0		16.00	525.00	898.21	0.95	1.24	0.39	45.45	0.97	29.18	10.04	100.52	33.18	0.10
511	84	1	20	1	1	3	1	1	1	0	0	0	0	0	21.8	6.00	343.00	2236.74	0.07	0.20	0.88	50.00	1.38	42.37	14.17	47.68	32.64	0.30
	84	2	20	1	1	3	1	1	1	0	0	0	0	0		7.00	391.00	62.34	0.37	1.24	0.38	40.91	1.23	37.29	12.40	32.22	32.97	0.38
512	85	1	19	2	1	3	1	1	1	0	0	0	0	0	20.4	11.00	367.00	751.50	0.57	1.24	1.36	47.73	1.29	37.30	16.53	45.10	34.61	0.37
	85	2	19	2	1	3	1	1	1	0	0	0	0	0		12.00	364.00	543.25	1.03	1.24	0.77	81.82	1.66	44.89	40.14	33.51	36.97	1.20
513	86	1	19	2	1	3	1	1	1	0	0	0	0	0	20.5	13.00	431.00	1394.73	0.11	0.20	0.88	43.18	0.95	28.24	8.26	38.02	33.74	0.22
	86	2	19	2	1	3	1	1	1	0	0	0	0	0		9.00	388.00	291.64	0.09	1.24	0.77	38.64	0.86	26.57	7.67	46.39	32.39	0.17
514	87	1	20	2	1	1	1	1	1	0	0	0	0	0	20.9	9.00	389.00	595.89	0.02	0.20	0.10	50.00	1.43	31.22	12.40	74.10	45.78	0.17
	87	2	20	2	1	1	1	1	1	0	0	0	0	0		9.00	373.00	19.92	0.22	1.25	0.16	70.45	2.04	37.74	25.38	79.25	54.17	0.32
515	88	1	20	2	1	3	1	1	1	0	0	0	0	0	16.2	12.00	437.00	1413.78	0.15	2.28	1.56	54.55	1.20	37.20	9.45	56.70	32.23	0.17
	88	2	20	2	1	3	1	1	1	0	0	0	0	0		12.00	495.00	574.75	0.10	1.24	0.77	77.27	1.15	37.95	20.66	40.59	30.37	0.51
516	89	1	20	2	1	3	1	1	1	0	0	0	0	0	19.2	6.00	346.00	970.62	0.07	0.20	0.58	52.27	1.40	48.11	31.88	34.79	29.07	0.92
	89	2	20	2	1	3	1	1	1	0	0	0	0	0		0.00	295.00	39.84	0.15	1.24	0.16	54.55	1.35	47.69	22.43	32.86	28.36	0.68
517	90	1	20	2	1	1	1	1	1	0	0	0	0	0	19.8	10.00	613.00	2304.06	0.39	0.20	1.76	61.36	1.34	46.36	31.88	152.71	28.84	0.21
	90	2	20	2	1	1	1	1	1	0	0	0	0	0		8.00	601.00	2148.34	0.09	1.24	0.99	56.82	1.66	51.00	30.70	179.12	32.54	0.17
518	91	1	20	1	1	2	1	1	1	0	0	0	0	0	22.5	7.00	527.00	1299.37	0.01	0.20	0.40	52.27	1.35	42.30	25.97	41.24	31.97	0.63
	91	2	20	1	1	2	1	1	1	0	0	0	0	0		4.00	578.00	1827.23	1.48	1.24	0.77	68.18	1.55	48.56	42.50	48.32	31.96	0.88
519	92	1	20	1	1	3	1	1	1	0	0	0	0	0	25.0	5.00	369.00	621.77	0.15	4.59	2.08	45.45	1.24	31.37	13.58	45.10	39.69	0.30
	92	2	20	1	1	3	1	1	1	0	0	0	0	0		2.00	385.00	945.23	0.51	1.24	1.54	88.64	2.01	30.86	23.61	52.84	65.24	0.45
